# Monitoring the level of government trust, risk perception and intention of the general public to adopt protective measures during the influenza A (H1N1) pandemic in the Netherlands

**DOI:** 10.1186/1471-2458-11-575

**Published:** 2011-07-19

**Authors:** Willemien van der Weerd, Daniëlle RM Timmermans, Desirée JMA Beaujean, Jurriaan Oudhoff, Jim E van Steenbergen

**Affiliations:** 1 Reigersbos 48, Amsterdam, 1106 AS, The Netherlands; 2 Department of Public and Occupational Health, VU University Medical Centre, Van der Boechorststraat 7, Amsterdam, 1081 BT, The Netherlands; 3 Centre for Infectious Disease Control, National Institute of Public Health and the Environment, Antonie van Leeuwenhoeklaan 9, Bilthoven, 3720 BA, The Netherlands

## Abstract

**Background:**

During the course of an influenza pandemic, governments know relatively little about the possibly changing influence of government trust, risk perception, and receipt of information on the public's intention to adopt protective measures or on the acceptance of vaccination. This study aims to identify and describe possible changes in and factors associated with public's intentions during the 2009 influenza A (H1N1) pandemic in the Netherlands.

**Methods:**

Sixteen cross-sectional telephone surveys were conducted (N = 8060) between April - November 2009. From these repeated measurements three consecutive periods were categorized based on crucial events during the influenza A (H1N1) pandemic. Time trends in government trust, risk perception, intention to adopt protective measures, and the acceptance of vaccination were analysed. Factors associated with an intention to adopt protective measures or vaccination were identified.

**Results:**

Trust in the government was high, but decreased over time. During the course of the pandemic, perceived vulnerability and an intention to adopt protective measures increased. Trust and vulnerability were associated with an intention to adopt protective measures in general only during period one. Higher levels of intention to receive vaccination were associated with increased government trust, fear/worry, and perceived vulnerability. In periods two and three receipt of information was positively associated with an intention to adopt protective measures. Most respondents wanted to receive information about infection prevention from municipal health services, health care providers, and the media.

**Conclusions:**

The Dutch response to the H1N1 virus was relatively muted. Higher levels of trust in the government, fear/worry, and perceived vulnerability were all positively related to an intention to accept vaccination. Only fear/worry was positively linked to an intention to adopt protective measures during the entire pandemic. Risk and crisis communication by the government should focus on building and maintaining trust by providing information about preventing infection in close collaboration with municipal health services, health care providers, and the media.

## Background

In 2009 a new influenza A (H1N1) virus began to spread in Mexico and the United States, causing the World Health Organization (WHO) to increase the pandemic alert level to phase 5 on April 29. This new virus spread rapidly to various countries over the world, and the possibility of a global pandemic neared when the alert level was raised to phase 6 on June 11 [1-7]. More than 214 countries had laboratory-confirmed cases of influenza A (H1N1), and the virus led to a total of 18.036 deaths by May 2010 [8].

In the early stages of a new pandemic influenza virus, there is usually no vaccine available. Other, non-medical, measures to control the spread of the disease such as the promotion of individual protection (face masks and hygiene), imposing travel restrictions, and social distancing of possibly infected cases are therefore essential [5,9-11]. The willingness of the public to comply with these kinds of measures, as proposed by public health authorities, is crucial to adequately control this type of event [5,6,10-15]. However, it remains rather difficult to motivate the public to actually institute recommended preventive measures [6,12,15]. Whether the public intends to adopt protective measures may be associated with their perceived risk of pandemic influenza [10,12,14-19] and their perception of the effectiveness of the government in dealing with such a crisis [9,13].

The Protection Motivation Theory suggests that a high level of risk perception can influence the intention of the general public to adopt protective measures. The theory states that perceptions of risk are determined by the public's perception of the severity of and their vulnerability to a certain health threat [14-23]. During an outbreak of a new pandemic influenza virus, information sources such as the government, public health professionals and the media, can inform the public about their vulnerability to the virus, the probability of contracting the disease, and its severity. Receiving such information via different avenues can affect the public's knowledge about their perceived risk, thereby influencing their decision to adopt protective measures [12-23]. It is therefore important to understand how the public perceives and to what degree they trust different sources that inform them about risks [4,6,12,16].

According to the Trust and Confidence Model, trust is an important factor in risk management because it can affect the public's judgments of risks and benefits, and can therefore indirectly influence the acceptance of recommended measures [24]. The model suggests that people with higher levels of trust or confidence in institutions, in this case the government, are more likely to accept recommended measures than those with lower trust or confidence levels [24]. Furthermore, trust can have a marked influence on risk perception, risk prevention behaviour, and government support [25,26]. It is central to how public health messages are heard, interpreted, and responded to [26]. Effective risk and crisis communication depends on how the public perceives and trusts the government during the course of a pandemic. A high level of public perception and trust is related to compliance with recommended measures [25,26]. Decreased trust in the government's ability to handle the threat may result from conflicting messages that can create scepticism about public health warnings [12,26].

Previous studies on government trust, risk perception and informational needs of the public were either conducted during times when pandemic influenza was only a hypothetical threat or when outbreaks were still in the relatively early stages [1,4,6,12-23,25,26]. In this present study data was not only gathered during the actual outbreak of the most recent pandemic influenza A (H1N1) virus, but multiple cross-sectional measurements (16) were conducted for an extensive period of time (April - November 2009). Besides, data was collected from a large, nationally representative sample. This study can therefore provide governments with a more thorough insight into the possibly changing reactions of the public during the course of a pandemic: not only about the public's level of government trust, but also about their level of risk perception, informational needs, and their willingness to adopt protective measures and accept vaccination. Unlike previous studies, this study can therefore add important knowledge to the field of emergency preparedness. Results can be used to develop future risk- and crisis communication methods and emergency preparedness plans, based on data that was collected during an actual pandemic flu.

This study was conducted during the 2009 influenza A (H1N1) pandemic in the Netherlands (April - November). The aim of the study was to identify and describe possible changes in the public's level of government trust, risk perception, and intention to adopt protective measures. Secondly, we wanted to identify whether government trust and risk perception were positively associated with an intention to adopt protective measures, including vaccination. Finally, we also hypothesised that receipt of information was positively associated with the public's intention to adopt protective measures, and with the acceptance of vaccination.

## Methods

### Study design, population and procedure

Commissioned by the Dutch Ministry of Health, the National Crisis Centre (NCC) performed 16 cross-sectional telephone surveys to monitor the level of government trust, risk perception, and intention to adopt protective measures during the influenza A (H1N1) pandemic. This resulted in a total study population of 8060 respondents aged 16 years and above. The 16 surveys were drawn, ad-hoc, from a large national panel of participants that were part of the risk and crisis panel of the consumer's panel of Market Response. This panel represents different geographical regions in the Netherlands, the so-called Nielsen regions. For each cross-sectional measurement different respondents were phoned at their registered home telephone number. To ensure a representative sample during a pending crisis, they could also be reached at their mobile number. Response rates varied from 52% to 73%, and the average time of conversation varied from 11 to 14 minutes. To avoid panel bias, only respondents who had not participated for at least one year in a previous survey were included up through measurement 15. For measurement 16, only respondents who had not participated for at least six months were eligible to participate. Because the study population was not comparable to the actual Dutch population, data were weighted for sex, age, education, family composition, and Nielsen region, in order to correct for differential non-response. The first survey took place on April 29, when the WHO raised the pandemic alert level to phase 5, and the sixteenth measurement was conducted on November 23, when the mass vaccination campaign in the Netherlands started (See Additional file 1). According to Dutch law the nature of this telephone survey, amongst healthy volunteers from the general population, does not require formal medical or ethical approval.

### Timing of the measurements in relation to the course of the influenza A (H1N1) pandemic

Based on a reconstruction of events regarding the epidemic in the Netherlands, three periods were determined: April - May, June - August, and August - November (See Additional file 1). During the first period, the WHO pandemic alert level was raised to phase 5. In the Netherlands, the epidemic was still in the relatively early phase and there was as yet no human-to-human spread. In the second period, the pandemic alert level was raised to phase 6, and at that time human-to-human spread occurred in the Netherlands. In August, the first death caused by the influenza virus was registered, and the government started its public health campaign. In the third period, the outbreak officially became an epidemic in the Netherlands. Four additional deaths were registered and general practitioners started to vaccinate risk groups. Also, the government's mass vaccination campaign started, directed at health care workers and children aged 1 - 5 years.

### Measurements

Because the questionnaire was drafted previously for use by the NCC, and was not based on a theoretical model, not all factors in the Trust and Confidence Model or the Protection Motivation Theory were measured. For the exact wording of the questions, see the questionnaire (See Additional file 2). For all variables, the category 'no opinion/do not know' was excluded from analyses.

#### Government trust

These concepts were measured on a scale from no trust at all (0) to a high level of trust (4) and were labelled according to the Trust and Confidence Model. For example, social trust encompassed trust in provided information, in measures already taken, and in fighting the pandemic. Overall trust, irrespective of crisis management, was labelled as a measure of past performance, and the perceived decisiveness of the government in taking safety measures was labelled as a measure of confidence. Factor analysis indicated that only one factor of the model was present (eigenvalue: 3.05), and therefore, the five measurements of government trust were summarized as one concept of trust (Cronbach's α: 0.832) on a scale of 0 to 15.

#### Risk perception

The level of fear was measured on a scale from not at all afraid (0) to very afraid (4), and worries about personal and family safety ranged from not worried at all (0) to very worried (3). These concepts were summarized as one concept of fear/worry about influenza A (H1N1) (Cronbach's α: 0.672) on a scale of 0 to 7. Perceived personal and family vulnerability ranged from absent (0) to very high (5).

#### Intention

Respondents were asked what kind of protective measures they intended to take, and answers were categorized as: 'no/do not know', 'hygienic measures', 'obtain medication/vaccination', or 'other measures' (scale 0 - 3). For the purpose of regression analysis, this variable was also dichotomized to range from 'no/do not know yet' to 'yes' (scale 0 - 1).

The intention to accept vaccination was analysed only during surveys 8 - 16 and was categorized as: 'no' (definitely not, probably not), 'do not know yet', and 'yes' (definitely, maybe) (scale 0 - 2). For the purpose of regression analysis, this variable was also divided into 'no' (definitely not, probably not, do not know yet) and 'yes' (definitely, maybe) (scale 0 - 1). Reasons to accept or refuse vaccination were also measured.

#### Information

Respondents were asked if they had received information about how to prepare for the influenza A (H1N1) pandemic (scale 0 - 1). They were also asked from which institutions they wanted to receive information, what kind of information they wanted, and why they did not trust the information provided by the government.

#### Demographic characteristics

These consisted of sex, age, educational level, family composition, and Nielsen region.

### Analysis

Estimates and 95% simultaneous (Bonferroni) confidence bands for the mean scores or proportions pertaining to government trust, fear/worry, perceived vulnerability, intention to adopt protective measures and to receive vaccination were computed along the three periods. These estimates help identify trends in various endpoints of interest. They are complemented by estimates and simultaneous (Bonferroni) 95% confidence intervals for the mean difference in scores or proportions from one period to the next. All confidence intervals presented are based on the normal approximation.

Forward logistic regression analyses were conducted for all periods. These analyses contained a basic model of government trust, fear/worry, and perceived vulnerability to define whether these variables were associated with the outcome measures: intention to adopt protective measures and intention to receive vaccination. Subsequently, receipt of information was added to the basic model to test for a possible association with both outcomes, followed by the addition of sex and age, education, family composition, and lastly, Nielsen region. Finally, backward logistic regression analyses were performed to test which of these variables were significantly associated with both outcomes (intention to adopt protective measures and intention to accept vaccination). Sex was analysed as a possible effect modifier with fear/worry and perceived vulnerability. P values < 0.05 were considered statistically significant, and data was analysed with SPSS version 18.0.

## Results

### Demographic characteristics

Weighted and unweighted demographic characteristics are presented in Table 1. Of the total weighted population (N = 8055), 50.8% were female, and the mean age of the population was 45.7 years (standard deviation [SD]: 17.69). Moreover, most respondents had received an intermediate level of education (41.5%), and most families did not include children (53.1%). The majority lived in the western regions of the Netherlands (28.9%).

**Table 1:** Weighted and unweighted demographic characteristics of respondents per time period

Characteristics	Weighted Total period (N = 8055)	Unweighted Period 1 (N = 3050)	Unweighted Period 2 (N = 2505)	Unweighted Period 3 (N = 2499)
**Sex (% female)**	50.8	57.3	60.6	53.6

**Age (mean/SD)**	45.7 (17.69)	50.42 (17.35)	50.19 (17.32)	49.83 (17.12)

**Education (%)**				

1. Low	29.5	30.4	32.5	29.4

2. Intermediate	41.5	37.5	37.6	36.9

3. High	29.0	32.1	29.9	33.7

**Family composition (%)**				

1. No children	53.1	57.3	57.2	54.6

2. Youngest child < 4 years	10.8	9.0	8.2	11.4

3. Youngest child > 5 years	36.0	33.8	34.5	33.9

**Nielsen region (%)**				

1. Amsterdam	5.2	3.8	3.6	3.4

2. Rotterdam	6.3	5.7	5.7	6.5

3. The Hague	4.1	3.3	3.1	3.2

4. West	28.9	28.7	28.6	29.8

5. North	10.5	12.1	13.5	13.1

6. East	20.9	21.6	20.2	19.7

7. South	24.2	24.8	25.3	24.4

SD = Standard Deviation

### Time trends in government trust, fear/worry, and perceived vulnerability

**Figure 1:**
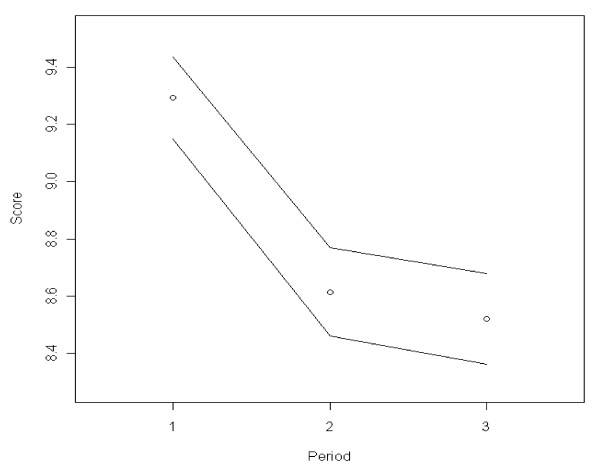
**Trends over time in government trust**. Black bordered white circle = Mean estimated score of government trust per time period. Black line = 95% confidence interval around the mean score.

During the course of the pandemic, the public's trust in the government decreased significantly from 9.29 in period one to 8.52 in period three (F [2.7810] = 70.05, p < 0.001) (Figure 1, Table 2). Results in Figure 1 consist of the plots of estimates of means per time period, with the corresponding confidence intervals. It shows that it is not possible to fit a horizontal line between bands of the first two graphs, indicating evidence of a decreasing trend in government trust (Figure 1, Table 2). However, on a scale of 0 to 15, trust remained relatively high.

**Table 2:** Trends over time in government trust, fear/worry, perceived vulnerability, an intention to adopt protective measures and to accept vaccination

	Period 1	Period 2	Period 3
1. Trust in government			
Mean (SD)	9.29 (2.66)*a*	8.61 (2.62)*b*	8.52 (2.64)*b*

MD with previous period	-	- 0.68	- 0.09

95% C.I. for MD with previous period	-	(0.84) - (0.52)	(0.26) - 0.07

*Scale 0 - 15*			

			

**2. Fear/worry about influenza A (H1N1)**			

Mean (SD)	2.11 (1.41)	2.10 (1.35)	2.01 (1.33)

MD with previous period	-	- 0.01	- 0.01

95% C.I. for MD with previous period	-	(0.09) - 0.07	(0.1) - 0.07

*Scale 0 - 7*			

			

**3. Perceived vulnerability**			

Mean (SD)	1.59 (0.97)*a*	2.31 (1.06)*c*	2.42 (1.09)*d*

Mean difference with previous period	-	0.72	0.11

95% C.I. for MD with previous period	-	0.66 - 0.78	0.04 - 0.18

*Scale 0 - 5*			

			

**4. Intention to adopt protective measures**			

Yes (%)	27.6a	45.0c	54.2d

MD with previous period	-	0.1	0.09

95% C.I. for MD with previous period		0.15 - 0.20	0.06 - 0.12

*Scale 0 - 1*			

			

**5. Intention to accept vaccination**			

Yes (%)	-	39.9	43.1

MD with previous period	-	-	0.03

95% C.I. for MD with previous period			0.00 - 0.06

*Scale 0 - 1*			

a: significant difference with period 2 - 3 (p < 0.001)

b: significant difference with period 1 (p < 0.001)

c: significant difference with period 1 - 3 (p < 0.001)

d: significant difference with period 1 - 2 (p < 0.001)

SD = standard deviation; MD = Mean difference; C.I. = Confidence Interval

**Figure 2:**
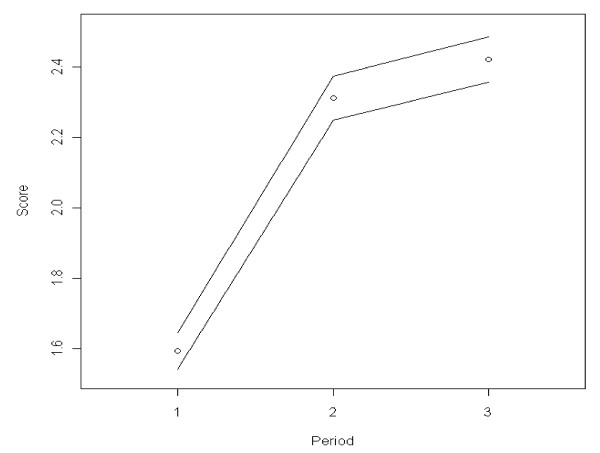
**Trends over time in perceived vulnerability**. Black bordered white circle = Mean estimated score of government trust per time period. Black line = 95% confidence interval around the mean score.

The public's perceived vulnerability to the influenza A (H1N1) virus increased significantly from 1.59 to 2.42 on a scale of 0 to 5 (F [2.7932] = 525.28, p < 0.001). Figure 2 shows evidence of an increasing trend in the public's perceived vulnerability overtime.

### Time trends in an intention to adopt protective measures, and in the acceptance of vaccination

**Figure 3:**
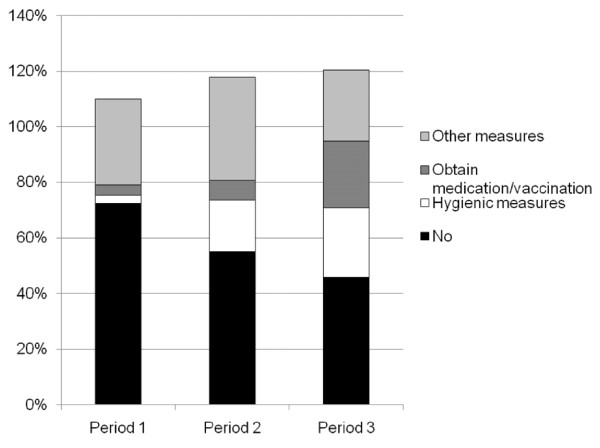
**Trends over time in an intention to adopt protective measures (multiple answers possible)**.

Intention to adopt protective measures increased significantly (F [2.8057] = 222.3, p < 0.001) from 27.6% in period one to 54.2% in period three (Table 2). Figure 3 shows which measures respondents intended to take during the course of the pandemic. An intention to adopt hygienic measures increased from 3% in period one to 25% in period three, and an intention to obtain medication/vaccination increased from 3.6% to 23.9%.

**Figure 4:**
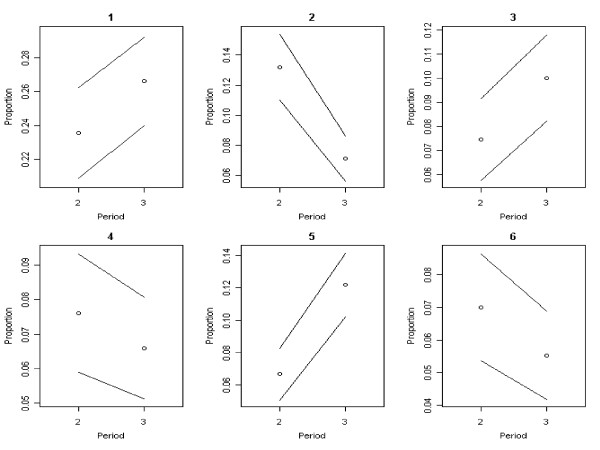
**Trends over time in reported reasons to refuse vaccination**. Black bordered white circle = Proportions of people who have given a reason for refusing vaccine Black line = 95% confidence interval around the proportion. Proportions for reasons 1 - 6 shown in this graph correspond with the meanings of reasons 1 - 6 in table 3.

**Figure 5:**
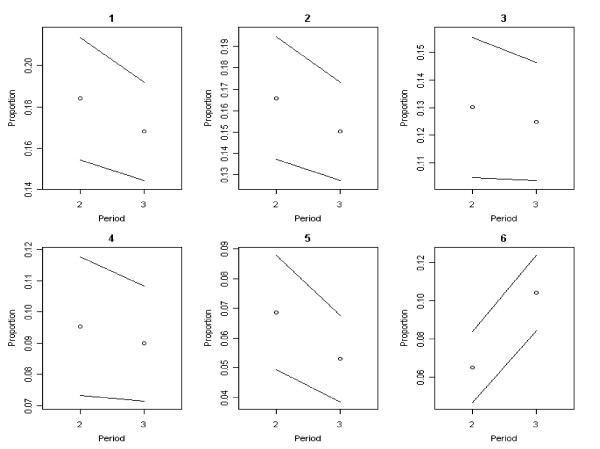
**Time trends in reported reasons to accept vaccination**. Black bordered white circle = Proportions of people who have given a reason for accepting vaccination. Black line = 95% confidence interval around the proportion. Proportions for reasons 1 - 6 shown in this graph correspond with the meanings of reasons 1 - 6 in table 3.

The most important reason to refuse vaccination was the mildness of the influenza (Table 3: reason 1). Mean differences in proportions between the periods show an increasing trend (Table 3, Figure 4: reason 1). Most respondents were willing to accept vaccination if persons close to them became sick or when their personal risk of infection or death would increase (Table 3). Mean differences in proportions between the periods indicate decreasing trends for all reasons (1 - 5) to accept vaccination, except for reason 6 which showed an increasing trend in the proportions of people saying they would never accept vaccination (Table 3, Figure 5). As the pandemic progressed, the percentage of respondents who still had doubts about vaccination decreased from 16.5% to 10.7%.

**Table 3:** Trends over time in reported reasons to accept or refuse vaccination (multiple answers possible)

	Period 2	Period 3	MD	95% C.I. for MD
**Reasons to accept vaccination (%)**				

1. When people close to me become sick	21.7	20.7	- 0.02	(0.04) - 0.01

2. When the risk of infection increases	19.3	18.3	- 0.02	(0.04) - 0.01

3. When the risk of death increases	15.9	14.8	- 0.01	(0.03) - 0.02

4. When it really becomes a disaster	11.6	10.7	- 0.01	(0.03) - 0.02

5. If the government advises it/if the vaccine is safe and effective	8.3/8.3	6.2/6.6	- 0.02	(0.03) - 0.00

6. I probably will not or will never be Vaccinated	8.1	12.2	0.04	0.02 - 0.06

				

**Reasons to refuse vaccination (%)**				

1. It is just a flu, not fatal, not necessary	28.5	33.1	0.030	0.00 - 0.06

2. Only if it is necessary	15.6	9.1	- 0.06	(0.08) - (0.04)

3. I do not or never get sick	8.9	12.3	0.03	0.00 - 0.04

4. I do not trust the vaccine	8.9	8.2	- 0.01	(0.03) - 0.00

5. I need more information	8.3	7.0	0.06	0.04 - 0.07

6. I am no risk group	8.0	14.2	- 0.01	(0.03) - 0.00

MD = Mean difference with previous period; C.I. = Confidence Interval

### Time trends in preferred information sources, need for and evaluation of government information

The percentage of respondents who had received information increased from 57.9% in period one to 85.2% in period three. During the course of the pandemic, the majority of respondents wanted to receive information from the municipal health services/health care providers (increase from 37.3% to 46.1%), and the media (increase from 24.5% to 30.0%). Other sources of information that were perceived as less important were general, local, and regional governmental institutions. Furthermore, respondents wanted information on how to prevent infection, what to do in the event of illness, symptoms, risks, consequences, and the number of infected cases. Trust in governmental information was high, but it decreased from 77.1% in period one to 61.0% in period three. At the start of the pandemic, the most reported reason to not trust governmental information was the perception that information was incomplete, kept secret or withheld (29.9%). In periods two and three the majority believed that the situation was exaggerated (26.9% and 30.2%). Other reported reasons were the perceptions that the government provided unclear information and that the government's information contradicted itself.

### Factors associated with an intention to adopt protective measures

Only in period one were all variables in the basic model (government trust, fear/worry, perceived vulnerability) significantly associated with an intention to adopt protective measures. Only fear/worry was significantly associated with this intention during all time periods. These associations remained after adjustment for demographic characteristics. At the start of the pandemic, higher levels of trust were associated with a lower intention to adopt protective measures. Respondents with higher levels of perceived vulnerability and fear/worry had a higher intention to adopt protective measures (Table 4). Furthermore, women's intention to adopt protective measures was higher than men's, and this increased with age. In period two, respondents who had received information, had a higher intention to adopt protective measures than those who did not receive information. The same effects of fear/worry, sex, and age on this intention were seen as in period one.

**Table 4:** Factors associated with an intention to adopt protective measures

	Wald (df)	OR	95% C.I. for OR
			**Lower - Upper**

**Period 1**			

1. Trust in government	24.86 (1)**	0.92	0.892 - 0.951

2. Fear/worry about influenza A (H1N1)	61.75 (1)**	1.30	1.218 - 1.389

3. Perceived vulnerability	41.42 (1)**	1.37	1.242 - 1.502

4. Sex (female)	6.97 (1)*	1.26	1.06 - 1.502

5. Age	32.78 (1)**	1.01	1.009 - 1.019

			

**Period 2**			

1. Fear/worry about influenza A (H1N1)	123.37 (1)**	1.44	1.354 - 1.541

2. Sex (female)	9.21 (1)*	1.31	1.100 - 1.557

3. Age	12.19 (1)**	1.01	1.005 - 1.017

4. Receipt of information (yes)	24.02 (1)**	1.65	1.352 - 2.020

			

**Period 3**			

*Men*			

1. Fear/worry about influenza A (H1N1)	53.17 (1)**	1.46	1.317 - 1.613

2. Age	33.43 (1)**	1.02	1.013 - 1.026

3. Receipt of information (yes)	5.48 (1)*	1.42	1.058 - 1.897

			

*Women*			

1. Fear/worry about influenza A (H1N1)	114.66 (1)**	1.74	1.573 - 1.927

2. Age	36.82 (1)**	1.03	1.017 - 1.033

3. Receipt of information (yes)	6.49 (1)*	1.73	1.134 - 2.632

* P - value significant at the < 0.05 level

** P - value significant at the < 0.001 level

df = degrees of freedom; OR = Odds Ratio; C.I. = Confidence Interval

Because results indicated that the effect of fear/worry on intention differed between men and women, the results were stratified for sex in period three. Women with a higher level of fear/worry had a higher level of intending to adopt protective measures than men. In addition, women who had received information were more likely to adopt measures than men that had also received information.

### Factors associated with an intention to accept vaccination

In periods two and three only fear/worry was significantly associated with an intention to receive vaccination in the basic model. However, after adjusting for demographic characteristics, both trust in the government and perceived vulnerability were significantly associated with this intention, as well. In both periods, higher levels of trust, fear/worry, and perceived vulnerability increased the intention to accept vaccination (Table 5). Also, respondents with intermediate or higher educational levels were less likely to intend to accept vaccination than those with less education. Family composition was associated with an intention to accept vaccination only in period two: families with children < 4 years of age or > 5 years were less likely to have this intention than those without children.

## Discussion

This is, to our knowledge, the first study that explores in multiple cross-sectional measurements the risk perception of the general population during an ongoing epidemic. The most important finding of this study was that higher levels of government trust were positively related to an intention to accept vaccination, but not to an intention to adopt protective measures (such as adopting extra hygienic precautions). From this perspective, another important outcome of our study was that, despite a significant reduction in trust during the overall study period, the level of trust still remained fairly high (mean score: 8.52 of 15 in period three). Also, recent European findings showed that Dutch residents experience the highest level of trust in the government, together with those from Luxembourg, Austria and Cyprus [27]. The statistically significant decrease in trust observed in our study might be explained by the initially presented worst-case scenario by the Dutch government. When this presented scenario was later followed by actual limited transmission, with limited morbidity and mortality, and the government's later statement that the virus was mild, this was translated and perceived as a conflicting governmental message [1]. It was shown earlier that government trust can decrease due to conflicting messages [12,26]. This might also clarify why we found that trust in information decreased: most respondents believed that information was withheld or kept secret from them in period one, while most believed that the government exaggerated the situation during periods two and three. This finding was also observed in other studies [1,7,28] and it shows the importance of effective risk and crisis-communication in maintaining and building trust in the government during a pandemic. Especially when high levels of trust are related to compliance with recommended measures that can control the spread of the disease [25,26]. Two other important results of this study were that an intention to adopt protective measures, as well as an intention to accept vaccination, increased during the course of the pandemic (from 27.6% in period one to 54.2% in period three and from 39.9% in period two to 43.1% in period three respectively). Also, higher levels of trust were positively related to an intention to accept vaccination. This study therefore adds important additional insights into the recently identified importance of finding governmental information reliable with having a strong intention to comply with measures in the near future, advised by that same government, by Bults et al [1]. Our study thereby further emphasizes the importance of building and maintaining trust in the government during an influenza pandemic, as was stated by the Trust and Confidence Model [24]. Quinn et al also confirms these results: during the H1N1 pandemic the acceptance of vaccination increased with higher levels of trust in the American government [4]. Importantly, the observed decline in trust in our study did not cause the public to be more afraid or worried [1]. Fear and worry remained stable and low during the entire pandemic. This is an appropriate reaction as the disease indeed presented itself as mild throughout the pandemic period [28]. The public's perceived vulnerability to the influenza A (H1N1) virus did increase, which can be related directly to the increased transmission rates in the Netherlands and the appearance of the first influenza related death in period two. These results are confirmed by other studies [1,6,7,28,29] and indicate that the Dutch general public had a clear understanding of the situation. In agreement with the Protection Motivation Theory and other studies conducted in the Netherlands, Australia, and the UK [1,2,6,13,21,28-31], our study found that higher levels of fear/worry and perceived vulnerability were positively linked to an intention to adopt protective measures and to an intention to accept vaccination. Interestingly, perceived vulnerability was only found to be a predictor of an intention to adopt protective measures, in period one. However, when fear and worry were not taken into account, vulnerability was positively associated with this intention. Our hypothesis that receipt of information would be related to a higher intention to adopt protective measures was established only in periods two and three. This is undoubtedly related to the absence of governmental information at the start of the pandemic and the fact that the government's public health campaign only focussed on vaccination in period three. In accordance with our study, Kok et al found that health care workers and municipal health services were perceived as the most trusted information sources during an influenza pandemic [1,28-32]. Information on how to protect oneself against infection was considered most urgent [28-32]. While Kok et al found that the media were the least trusted source of information [27], our study indicates that they were the second most important source of information during the course of this pandemic. Not only did this study analyse data from a governmental self-evaluation, it was also conducted during the threat of an actual new influenza pandemic. It therefore provides important insights into the reactions of the public, in addition to the number of cross-sectional and laboratory and hypothetical studies. Moreover, trends over time could be identified due to repeated measurements. This is an important improvement over previous studies that either consisted of one cross-sectional survey or that were only conducted in the early phase of the pandemic. In addition to the previously conducted, smaller, Dutch study by Bults et al using an internet panel [1], we showed similar time trends in the public's fear and worry, but we were able to relate this to trust in the government. Because this study did not include follow-up data (cross-sectional measurements were not taken from the same group of respondents), changes over time might not have reflected real time trends. Nevertheless, this study is, to our knowledge, the only one that was able to determine the influence of government trust on intentions of the general public to adopt protective measures for an extensive period of time and amongst a large study population (N = 8055). Results of our study can therefore have important implications for effective health-risk communication by the government during future influenza pandemics. However, because the questionnaire was not based on a theoretical model, it did not encompass all the variables that should be measured to effectively predict intention in accordance with the Trust and Confidence Model or the Protection Motivation Theory. Also, this study might not reflect actual behaviour, since only intentions of the public were measured. The preferred study would include an intervention and a control group. In pandemic or other outbreak situations it is ethically-challenging however, to advocate a study where certain groups do not receive the best possible information.

**Table 5:** Factors associated with an intention to accept vaccination

	Wald (df)	OR	95% C.I. for OR
			**Lower - Upper**

**Period 2**			

1. Trust in government	5.20 (1)*	1.05	1.006 - 1.088

2. Fear/worry about influenza A (H1N1)	109.31 (1)**	1.53	1.416 - 1.662

3. Perceived vulnerability	7.34 (1)*	1.15	1.040 - 1.279

4. Age	35.25 (1)**	1.02	1.015 - 1.029

5. Education (Low)	11.13 (2)*		

*Intermediate*	5.44 (1)*	0.75	0.584 - 0.954

*High*	10.69 (1)**	0.64	0.486 - 0.835

6. Family composition (No children)	10.68 (2)*		

*Youngest child < 4 years*	5.92 (1)*	0.63	0.437 - 0.915

*Youngest child > 5 years*	8.86 (1)*	0.68	0.524 - 0.875

7. Nielsen region (Amsterdam)	19.15 (6)*		

*Rotterdam*	1.23 (1)	0.72	0.408 - 1.282

*The Hague*	0.72 (1)	1.32	0.699 - 2.478

*West*	3.10 (1)	0.66	0.421 - 1.048

*North*	8.34 (1)*	0.46	0.273 - 0.780

*East*	5.90 (1)*	0.56	0.346 - 0.893

*South*	3.04 (1)	0.66	0.416 - 1.053

**Period 3**			

1. Trust in government	8.81 (1)*	1.05	1.018 - 1.089

2. Fear/worry about influenza A (H1N1)	69.80 (1)**	1.36	1.265 - 1.461

3. Perceived vulnerability	4.71 (1)*	1.10	1.009 - 1.200

4. Age	199.82 (1)**	1.04	1.035 - 1.046

5. Education (Low)	16.93 (2)**		

*Intermediate*	4.24 (1)*	0.80	0.641 - 0.989

*High*	16.88 (1)**	0.62	0.487 - 0.775

* P - value significant at the < 0.05 level

** P - value significant at the < 0.001 level

df = degrees of freedom; OR = Odds Ratio; C.I. = Confidence Interval

## Conclusions

Despite intense media coverage, the response of the Dutch general public to the influenza A (H1N1) virus was relatively muted. Our study showed the importance of government trust and risk perception with respect to vaccination acceptance. More research is needed to investigate these relationships during future pandemics. Because effective health-risk communication is important to protect public health during a pandemic [26], this study's outcomes are important for governmental risk and crisis communication. The government should maintain trust by providing the public with complete pandemic information during its entire course, even when knowledge is limited. Importantly, to ensure clear public understanding of the situation, the government must provide information about risk, existence and effectiveness of preventive actions or measures, and safety and efficacy of vaccination. It is advised not to downplay the actual risk and vulnerability in the hope to reduce the public's fear and worry. Information should be composed and presented in close collaboration with municipal health services, health care providers, and the media to effectively reach the public. This will build and maintain trust in the government and will increase vaccination uptake, as well as adherence to other recommended measures that can control the spread of the disease.

## Competing interests

The authors declare that there have been no competing interests.

## Authors' contributions

WvdW searched the literature, contributed to the formation of the study and the manuscript, performed the analyses, and interpreted the data. DRMT substantially contributed to the formation of the study, methodology, and interpretation of the data. DJMAB and JEvS initiated and contributed to the study and the interpretation of the data. JO made substantial contributions to the methodology and interpretation of the data. All authors read and approved the final manuscript.

## Pre-publication history

The pre-publication history for this paper can be accessed here:

http://www.biomedcentral.com/1471-2458/11/575/prepub

## Supplementary Material

Additional file 1 **Description of events during the influenza A (H1N1) pandemic per time period**. This description provides an overview of the dates of the sixteen telephone surveys. A more thorough description of the classification of the three time periods is given, related to important events during the influenza A (H1N1) pandemic. Click here for file

Additional file 2 **Survey questions**. These survey questions were used across the sixteen telephone surveys and were used for data-analysis. Click here for file
