# Supplementary material for: Monitoring the level of government trust, risk perception and intention of the general public to adopt protective measures during the influenza A (H1N1) pandemic in the Netherlands
Source: BMC Public Health. 2011 Jul 19;11:575. doi: 10.1186/1471-2458-11-575 (PMC3152536; doi:10.1186/1471-2458-11-575)
Supplement: Additional file 1 — Description of events during the influenza A (H1N1) pandemic per time period. This description provides an overview of the dates of the sixteen telephone surveys. A more thorough description of the classification of the three time periods is given, related to important events during the influenza A (H1N1) pandemic. [file 1471-2458-11-575-S1.DOC]

**Additional file 1. Description of events during the influenza A (H1N1) pandemic per time period**

These are the dates of the 16 telephone surveys performed between April – November 2009:

Survey 1: April 29 Survey 2: May 1 Survey 3: May 4 Survey 4: May 6 Survey 5: May 8 Survey 6: May 25 Survey 7: June 22 Survey 8: August 3 Survey 9: August 17 Survey 10: August 31 Survey 11: September 14 Survey 12: September 28 Survey 13: October 12 Survey 14: October 26 Survey 15: November 9 Survey 16.: November 23

These cross-sectional measurements were classified into three time periods based on a reconstruction of facts during the influenza A (H1N1) pandemic in the Netherlands.

**Period 1: 29 April 29 – 25 May (Survey 1 – 6)**

On April 29 the WHO raised the pandemic alert level to phase 5, because there was evidence that the virus could spread between humans. At this point, the influenza epidemic was still in the early, relatively stable phases in the Netherlands. Of the three cases of influenza, all of the patients became infected in Mexico, indicating that there was at that point no human-to-human spread in the Netherlands.

Between period 1 and two, the total number of confirmed cases in the Netherlands increased, as well as the number of patients infected by secondary transmission. On June 11 the WHO raised the pandemic alert level to phase 6, implying that a global pandemic was close, and the Ministry of Health ordered 34 million doses of vaccine to protect the entire Dutch population.

**Period 2: 22 June – 14 September (Survey 7 – 11)**

During the second period the first death occurred (4 August), and the Health Council advised vaccinating only risk groups (17 August). On August 19 the government started their public health campaign, ‘Fight the Flu’, and on September 10 an article was published that raised concern among the population, because it implied that one dose of the vaccine was sufficient, making a second dose unnecessary.

Between periods 2 and 3, the Health Council changed its advice (17 September) and recommended vaccinating pregnant women without a medical indication. According to the Health Council, it was not necessary to vaccinate healthy children, and it emphasized that one dose of the vaccine was not sufficient to fully protect the population. In addition, a second death occurred on September 27.

**Period 3: 28 September – 23 November (Survey 12 – 16)**

During the third period, an epidemic became official in the Netherlands. Four more people died, and in October, the death of a healthy girl heightened media attention and concern among the population. In addition, many questions were raised by the public about certain components of the vaccine. On November 2, general practitioners started vaccinating risk groups, and on November 9, the Health Council advised also vaccinating children between six months and five years of age, as well as those living with infants up to the age of 6 months. Pregnant women were advised to be vaccinated from their second trimester. On November 23, the mass vaccination campaign started.
