# Supplementary material for: Monitoring the level of government trust, risk perception and intention of the general public to adopt protective measures during the influenza A (H1N1) pandemic in the Netherlands
Source: BMC Public Health. 2011 Jul 19;11:575. doi: 10.1186/1471-2458-11-575 (PMC3152536; doi:10.1186/1471-2458-11-575)
Supplement: Additional file 2 — Survey questions. These survey questions were used across the sixteen telephone surveys and were used for data-analysis. [file 1471-2458-11-575-S2.DOC]

**Additional file 2. Survey questions**

| **Items labelled according to the Trust and Confidence Model** | | |
| --- | --- | --- |
| ***Question*** | ***Concept*** | ***Scale*** |
| During a major crisis, the government informs you about the concerned crisis. How much trust do you generally have in information provided by the government about the Mexican flu? | Social trust | 0 – 4 |
| How much trust do you have in measures already taken by the government against the Mexican flu? | Social trust | 0 – 4 |
| How much trust do you have in the government with respect to fighting the pandemic? | Social trust | 0 – 4 |
| What do you think of the decisiveness of the government in taking safety measures against the Mexican flu in the Netherlands? | Confidence | 0 – 4 |
| How much trust do you generally have in the government, irrespective of crisis management? | Past performance | 0 – 4 |

| **Items labelled according to the Protection Motivation Theory** | | |
| --- | --- | --- |
| ***Question*** | ***Concept*** | ***Scale*** |
| To what extent are you afraid that an epidemic in humans will occur in the Netherlands?* | Fear | 0 – 4 |
| To what extent are you currently worried about your personal and family safety due to the Mexican flu in the Netherlands?* | Worry | 0 – 3 |
| What do you think are the chances you or your family will be infected with the Mexican flu? | Perceived vulnerability | 0 – 5 |

** For the purpose of analysis fear and worry were analysed as one concept of fear/worry on a scale of 0 – 7*

**Questions related to informational needs of the Dutch study population**

**Question 7.0:** Why do you have complete trust/no trust at all in current information?

1. Little or no information received. 2. Colored, one-sided information, propaganda. 3. Information withheld, kept secret, information not complete. 4. Unclear information. 5. No trust in information from the government. 6. Government does not take citizens seriously. 7. No trust in government/politics. 8. No trust in the government in itself. 9. Information was too late. 10. Government does not keep up track of the facts. 11. Communication failure. 12. Not focused on information, but on prevention of panic. 13. Government underestimates the problem. 14. Government often contradicts herself. 15. It is exaggerated. 16. Government does not have (enough) knowledge. 17. Economic issue. 98. Other reasons. 99. Do not know.

**Question 9.0:** Have you recently seen, heard or read something about what to do in case of the Mexican flu?

1. Yes. 2. No. 3. Do not know.

**Question 11.0:** By whom would you mostly want to be informed about the Mexican flu?

1. Police department. 2. Fire department. 3. Mayor. 4. Municipal health services/health care workers. 5. National Institute for Public Health and the Environment. 6. Local government, municipality. 7. Regional government. 8. Head public safety. 9. Prime minister. 10. Ministry of Home Affairs. 11. Ministry of Foreign Affairs/ general ministries. 12. General government (not specified). 13. PostBus 51. 14. Expert/virologist/epidemiologist. 15. Radio. 16. Television. 17. Internet. 18. Papers. 19. Local media. 20. Media. 21. Ministry of Public Health. 22. Work/employer. 23. Does not matter. 24. Queen. 25. Family/friends. 26. World Health Organization. 27. School. 28. Insurance company. 29. Government. 96. I do not want to be informed. 98. Other. 99. Do not know.

**Question 13.0:** What kind of information would you like to receive about the Mexican flu?

1. Symptoms/how do you know that you have the Mexican flu? 2. Current situation: number of cases. 3. Current situation: distribution of cases. 4. What to do when you have the Mexican flu/reporting. 5. Prevention/protection/precautionary measures. 6. Vaccination/inoculation. 7. Risks and consequences. 8. General information. 9. Current situation (not specified). 10. Information about the virus. 11. What is the government doing? 12. Progression of the disease. 13. What is the effect for the risk group? 14. Objective/honest information. 90. No information needed. 96. Do not want to be informed. 98. Other. 99. Do not know.

**Questions related to an intention to adopt protective measures of the Dutch study population**

**Question 21.0:** Do you personally intend to take measures to protect yourself and your family against the Mexican flu? (Five possible answers)

1. No. 2. Avoid (physical) contact. 3. Follow recommended advice. 4. Avoid contact with possibly infected persons. 5. Hygiene. 6. Look for information. 7. Obtain medicines/vaccination. 8. Face mask. 9. Avoid travelling. 10. What can I do? (I do not have enough information). 11. Yes, if there are measures. 12. Live healthy. 13. Visit a physician. 14. Stay alert. 15. Remain inside. 16. Yes, if needed. 17. Avoid large groups of people. 95. Yes, namely: Type in open column. 98. Other. 99. Do not know yet.

**Question 28.0:** Do you intend to receive vaccination against the Mexican flu?

1. Yes, definitely. 2. Yes, maybe. 3. I do not know yet. 4. No, probably not. 5. No, definitely not.

**Question 29.0:** Why are you not sure yet? Why do you (probably) not want to receive vaccination? (Four possible answers)

1. I will not get/am never sick. 2. It is just a flu/not fatal/ not necessary. 3. I never get vaccinated against the flu. 4. I do not trust the vaccine. 5. I do not want to pay for it. 6. Only if it is necessary. 7. Only if it comes close to me. 8. It depends on the risks/adverse events. 9. Vaccination is worse than the flu. 10. I have not thought about it yet. 11. I am not in a risk group. 12. I need more information first. 13. There is no medicine yet. 14. I already get a flu shot. 15. Only if it is necessary. 16. I already received it. 17. It does not help. 18. Depends on employer. 95. Other reason, namely: type in open column. 98. Other. 99. Do not know yet.

**Question 30.1:** Under which circumstances would you definitely want to receive vaccination? (Four possible answers)

1. I will probably not/will never get vaccinated. 2. If people around me/close to me become sick. 3. When the chance of infection increases. 4. When the chance of death increases. 5. When I am obligated according to physicians. 6. When I am obligated according to the government.

7. When it really becomes a disaster. 8. When the vaccine is effective and safe. 9. When I am obligated according to the employer.

10. Depends on the information. 11. When I belong to a risk group. 12. When I would become sick. 13. If I am pregnant.

14. When it is possible. 95. Other reason, namely: type in open column. 98. Other. 99. Do not know yet
